# A Stem Cell-Based Tool for Small Molecule Screening in Adipogenesis

**DOI:** 10.1371/journal.pone.0013014

**Published:** 2010-09-27

**Authors:** Jie Qin, Wei-Qiang Li, Li Zhang, Fei Chen, Wen-Hua Liang, Frank Fuxiang Mao, Xiu-Ming Zhang, Bruce T. Lahn, Wei-Hua Yu, Andy Peng Xiang

**Affiliations:** 1 Center for Stem Cell Biology and Tissue Engineering, Sun Yat-Sen University, Guangzhou, Guangdong, People's Republic of China; 2 The Key Laboratory for Stem Cells and Tissue Engineering, Ministry of Education, Guangzhou, Guangdong, People's Republic of China; 3 Howard Hughes Medical Institute, Department of Human Genetics, University of Chicago, Chicago, Illinois, United States of America; 4 Zhongshan Medical School, Sun Yat-sen University, Guangzhou, Guangdong, People's Republic of China; 5 Cell Therapy Center, The Third Affiliated Hospital, Sun Yat-sen University, Guangzhou, Guangdong, People's Republic of China; University of Tor Vergata, Italy

## Abstract

Techniques for small molecule screening are widely used in biological mechanism study and drug discovery. Here, we reported a novel adipocyte differentiation assay for small molecule selection, based on human mesenchymal stem cells (hMSCs) transduced with fluorescence reporter gene driven by adipogenic specific promoter - adipocyte Protein 2 (aP2; also namely Fatty Acid Binding Protein 4, FABP4). During normal adipogenic induction as well as adipogenic inhibition by Ly294002, we confirmed that the intensity of green fluorescence protein corresponded well to the expression level of aP2 gene. Furthermore, this variation of green fluorescence protein intensity can be read simply through fluorescence spectrophotometer. By testing another two small molecules in adipogenesis –Troglitazone and CHIR99021, we proved that this is a simple and sensitive method, which could be applied in adipocyte biology, drug discovery and toxicological study in the future.

## Introduction

Adipogenesis is a process of adipocyte formation. Adipocytes in white adipose tissue are crucial for maintaining energy balance and body homeostasis [1]. White fat can secrete different kinds of adipokines to regulate many activities, including glucose and lipid metabolism, inflammatory responses, angiogenesis and reproduction throughout the body [2]. Dysfunction of this cell population would lead to many kinds of metabolic diseases, such as cardiovascular diseases and type 2 diabetes [3], [4]. Understanding the mechanisms involved in adipogenesis might add novel insights to the adipose tissue development, as well as therapies of metabolic diseases. To this end, potent and specific small molecule regulators in adipogenesis might serve as powerful research tools. Researchers have made many efforts to screen different kinds of small molecules to study the molecular mechanisms of adipogenic differentiation and for drug discovery [5], [6], [7].

At present, there are many methods utilized in differentiation assays to screen small molecules, including luciferase reporter assay, antibody label assay and fluorescence protein assay [8], [9], [10], [11], [12], [13]. One study screened 500 compounds from a small-molecule library for activators and repressors in adipogenesis by using aP2Luc-3T3F442A reporter cell line [6]. However, murine cell lines are not ideal models to study human adipogenesis, due to the genetic profiling differences from human cells. Human mesenchymal stem cells derived from bone marrow can differentiate into osteocytes, adipocytes and chondrocytes, and maintain this multipotency during in vitro culture [14], [15]. Moreover, hMSCs-derived adipocytes have been proved to be morphologically and functionally identical to human mature fat cells [16]. Therefore, hMSCs are considered as a useful differentiation model to unravel the molecular events involved in human adipogenesis. In this study, we introduce a simple and sensitive system using hMSCs transduced with aP2 promoter driving hrGFP reporter gene for screening small molecules in adipogenesis.

## Results

### Generation of the aP2-hrGFP hMSC reporter cell line

**Figure 1:**
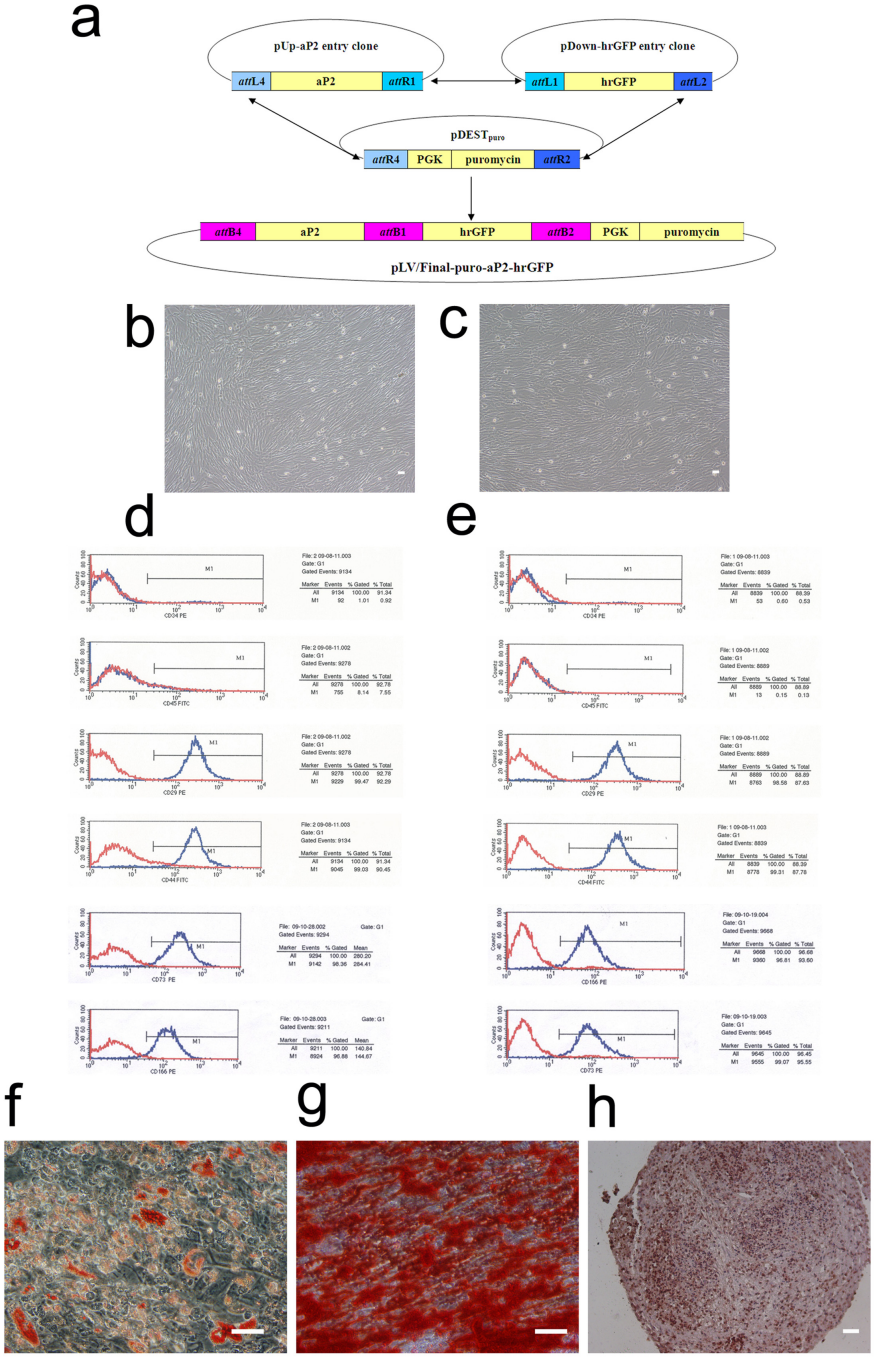
Vector construction and lentivirus transduction, characterization, and differentiation of hMSCs. a, Construction of pLV/Final-puro-aP2-hrGFP; b, Phase contrast microscopy of hMSCs at Passage 9, one day before transduction; c, Phase contrast microscopy of hMSCs at Passage 11 after puromycin selection; d, Flow-cytometric analysis of cell surface antigens of untransduced hMSCs; e, Flow-cytometric analysis of cell surface antigens of transduced hMSCs; f, Oil red O staining of adipogenic differentiated transduced hMSCs; g, Alizarin red S staining of osteogenic differentiated transduced hMSCs; h, Collagen II staining of chondrogenic differentiated transduced hMSCs. Bar = 50 µm.

To establish a stem cell-based system for small molecule screening, we generated the human mesenchymal stem cell line transduced with aP2-hrGFP lentivector. The expression clones, pLV/Final-puro-aP2-hrGFP (Fig. 1a) was generated as described in methods. After virus transduction and antibiotic selection, purified aP2-hrGFP hMSCs were attained. Observing under the phase-contrast microscope, there was no significant morphological difference between the untransduced and transduced hMSCs (Fig. 1b, 1c). Additionally, in order to make better advantage of the multipotency of hMSCs, we double transduced these cells with two vectors containing adipocyte specific promoter (aP2) and osteoblast specific promoter (2.3Col, collagen type I 2.3 kb) [17] respectively, and obtained purified aP2-hrGFP/2.3Col-RFP hMSCs after antibiotics selection, which can be used to screen compounds for effects on adipogenic and osteogenic lineages simultaneously. (Fig. S1, a, b)

### Characterization of aP2-hrGFP hMSCs

To examine the characteristics of aP2-hrGFP hMSCs, we used FACS to analyze cell surface markers of hMSCs. Compared with untransduced hMSCs, transduced cells expressed the same panel of known markers, including CD29, CD44, CD73, CD166, but did not express specific markers of hematopoietic stem cells, such as CD34 and CD45 (Fig. 1d, 1e), which indicated that transduced cells maintained the phenotype of hMSCs.

Furthermore, to demonstrate the multipotency of aP2-hrGFP hMSCs, cells were subjected to differentiate into adipo-, osteo-, and chondrogenic lineages. By oil red O staining, lipid droplets can be observed in the cytoplasm of adipocytes on Day 15 of adipogenic differentiation (Fig. 1f). After 15 days of culture in osteogenic medium, hMSCs differentiated into osteoblasts, which were confirmed by strong alizarin red S staining (Fig. 1g). Chondrogenic differentiation was confirmed by immunohistochemical analysis, which showed the presence of human type II collagen (Fig. 1h).

### Verification of aP2 promoter controlled cell-based system

To verify the aP2-hrGFP hMSCs selection system, we first performed normal adipogenic experiment. In order to determine the most suitable time point for detection, we performed adipogenic induction for 30 days. We used real-time PCR and fluorescence spectrophotometer to analyze the variation of aP2 gene expression and hrGFP intensity among different differentiated time points. The results indicated that both the aP2 gene expression and hrGFP intensity peaked on D18 of adipogenic induction, and decreased in later time period (Fig. S2, a, b). However, D18 differentiated cells showed less cell viability and had the similar lipid droplet accumulation as D15 differentiated cells (Fig. S2, c–e). As a result, we believe that it is much more suitable to detect aP2 gene expression and hrGFP intensity on D15 of differentiation, rather than D18 of differentiation.

**Figure 2:**
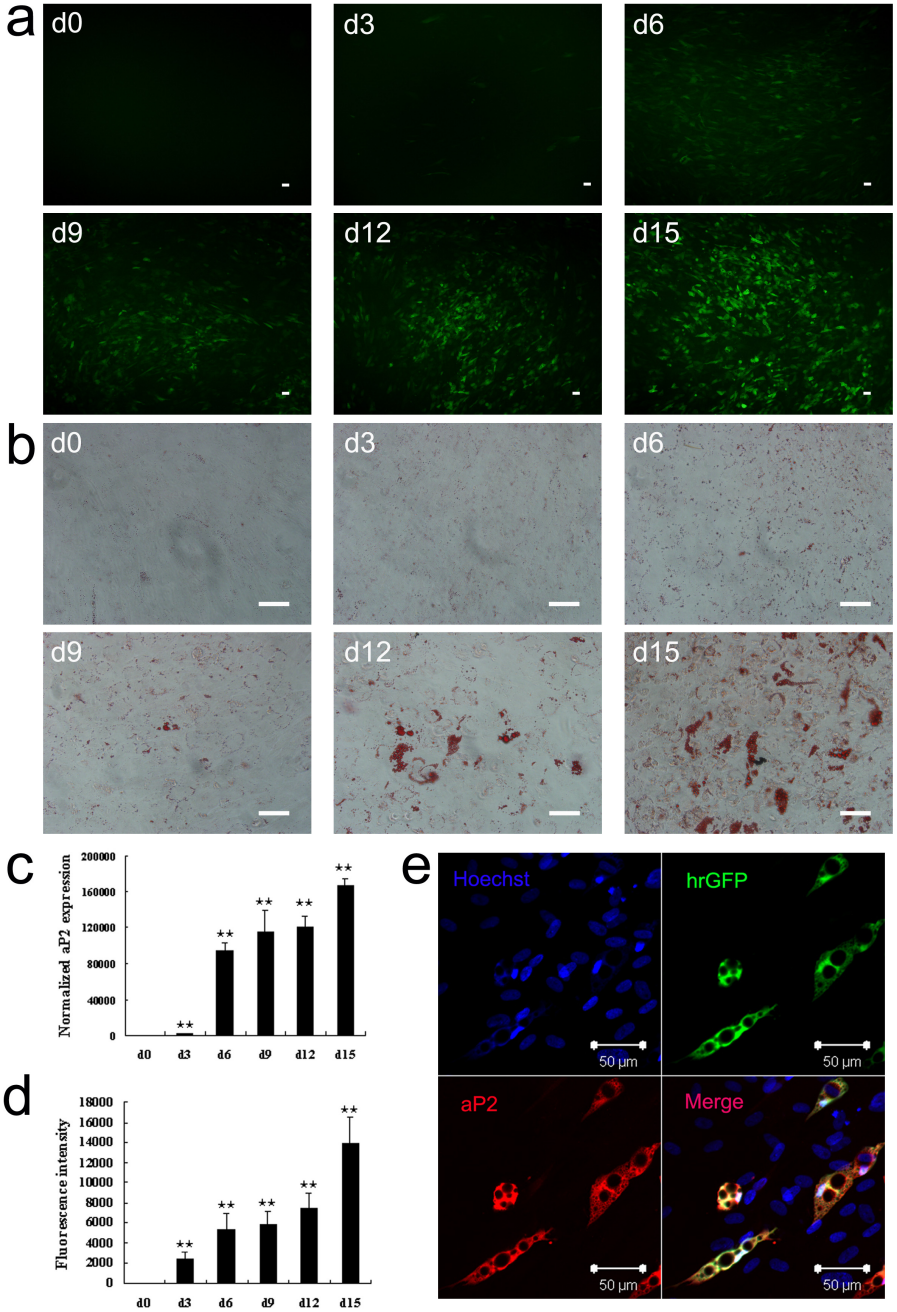
Normal adipogenic induction of aP2-hrGFP hMSCs. a, hrGFP expression of aP2-hrGFP hMSCs in different days of adipogenesis; b, Oil red O staining results of aP2-hrGFP hMSCs in different days of adipogenic differentiation; c, Normalized mRNA expression of aP2 gene determined by real-time PCR analysis during adipogenesis; d, Fluorescence intensity detection of aP2-hrGFP hMSCs by fluorescence spectrophotometer during normal adipogenic induction; Data are presented as mean ± SD of three independent experiments, and the p-values in the graph show the statistical significance of the difference between each test day and Day 0. (^★^ *p*<0.05; ^★★^ *p*<0.01). e, Immunofluorescence staining of aP2 protein on Day 15 of adipogenic induction (e). Bar = 50 µm.

Next, during 15 days of adipogenic induction, we observed the hrGFP gene expression every three days through fluorescence microscope and performed the oil red O staining for comparison. Also, we detected the fluorescence intensity by fluorescence spectrophotometer at the same time points, and together with detection of aP2 gene expression by real-time PCR. The results showed that the expression levels of fluorescence protein concurred with the lipid droplet accumulation (Fig. 2a, 2b). The fluorescence intensity increased in a time-dependent manner, corresponding to the elevation of aP2 gene expression (Fig. 2c, 2d). And then, we performed immunofluorescence staining with anti-aP2 antibody, which demonstrated that aP2 and hrGFP genes were co-expressed in adipocytes (Fig. 2e).

To further prove the feasibility of this system, we treated aP2-hrGFP hMSCs with LY294002 - an inhibitor of Phosphatidylinositol (PI) 3-kinase (PI3K) pathway [18]. PI3K signaling pathway is involved in the early stage of adipogenesis. Inhibition of PI3K pathway would inhibit the mRNA expression of PPAR gamma and LPL, which are essential genes in the process of adipogenic differentiation [19].

**Figure 3:**
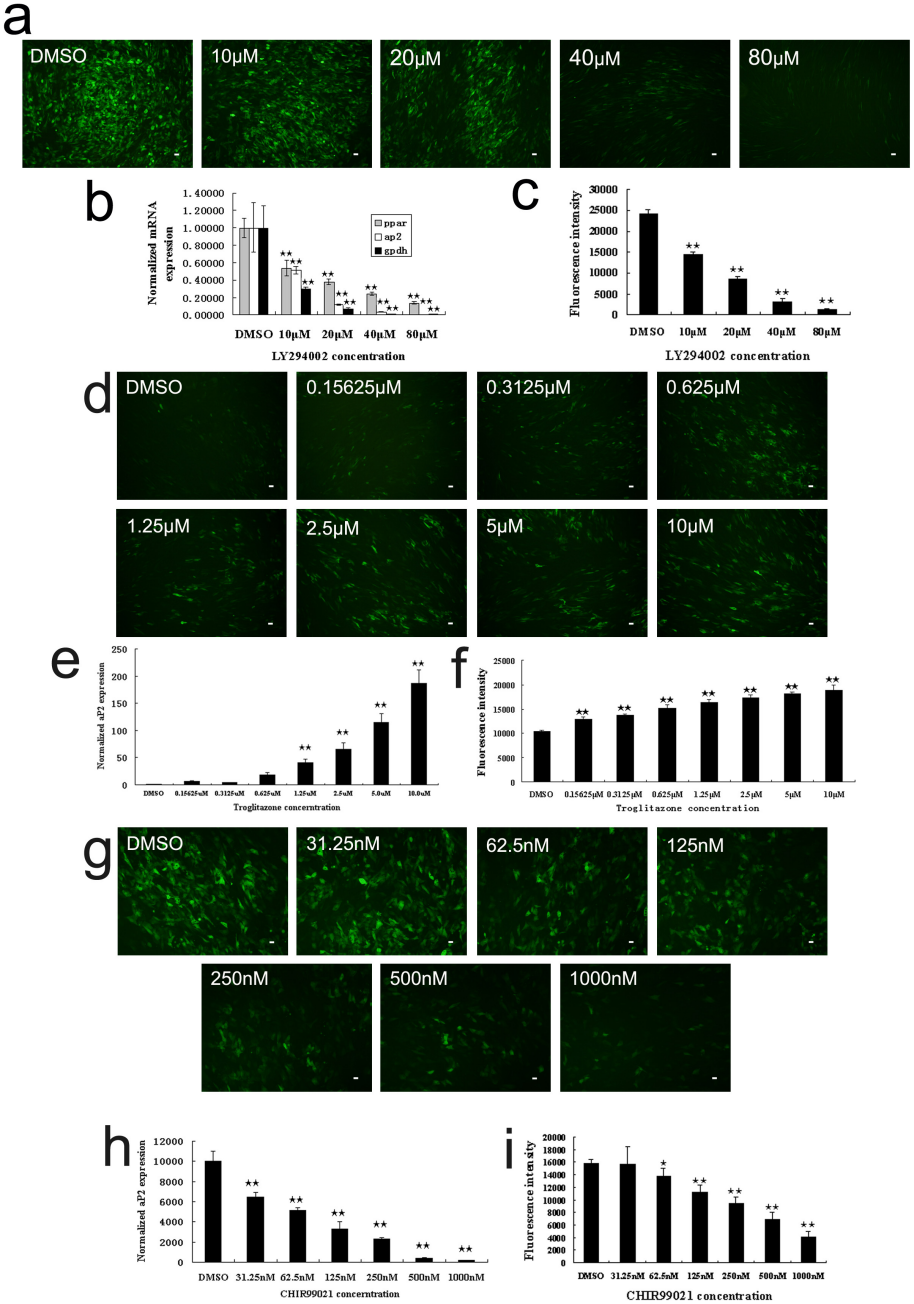
Adipogenic differentiation assays of aP2-hrGFP hMSCs with LY294002 (a–c), Troglitazone (d–f) and CHIR99021 (g–i). a, Fluorescence observation of LY294002 assay with different concentrations, after 15 days of adipogenic induction; b, Normalized mRNA expression of PPAR gamma, aP2 and GPDH genes determined by real-time PCR in LY294002 assay; c, Fluorescence intensity detection of LY294002 assay on Day 15 of differentiation; d, Fluorescence observation of aP2-hrGFP hMSCs with different concentrations of Troglitazone, on Day 15 of differentiation; e, Normalized mRNA expression of aP2 gene determined by real-time PCR in Troglitazone assay; f, Fluorescence intensity detection of Troglitazone with relative concentrations, after 15 days of adipogenic induction; g, Fluorescence observation of aP2-hrGFP hMSCs with different concentrations of CHIR99021, on Day 15 of differentiation; h, Normalized mRNA expression of aP2 gene determined by real-time PCR in CHIR99021 assay; i, Fluorescence intensity detection of CHIR99021 with relative concentrations, after 15 days of adipogenic induction. Data are presented as mean ± SD of three independent experiments, and the p-values in the graph show the statistical significance of the difference between each test group and the DMSO control group. (^★^ *p*<0.05; ^★★^ *p*<0.01). Bar = 50 µm.

The tests were performed in 1∶2 serial dilutions, with 80 µM, 40 µM, 20 µM and 10 µM final concentrations, respectively (with the same concentration of the solvent DMSO (0.16%) in all groups) (Fig. 3a). After 15 days of induction, the 96-well test plates were read through the fluorescence spectrophotometer. Besides, RNA samples of Day 15 differentiated cells were collected, and we used real-time PCR for quantitative detection of three adipogenic specific genes expression – PPAR gamma, aP2 and GPDH. By comparing the data from the fluorescence spectrophotometer with that from real-time PCR, we showed that the results of fluorescence spectrophotometer were in consistent with that of real-time PCR (Fig. 3b, 3c). This finding demonstrated that the aP2 gene expression was closely co-related with hrGFP gene expression from another aspect, and the fluorescence protein assay was as sensitive as real-time PCR. Additionally, from the data of fluorescence spectrophotometer, we found that the inhibition effect of LY294002 acted in a dose-dependent manner, with the effective concentration for half-maximum response (EC_50_) of 15 µM and the maximum effective concentration of 40 µM.

### Tests of other small molecules

To find out whether this system can also be applied in detection of other small molecules, we treated aP2-hrGFP hMSCs with one agonist and one inhibitor of adipogenesis - Troglitazone and CHIR99021.

Troglitazone is an agonist for PPAR gamma. It can stimulate adipogenesis by elevating adipogenic specific genes expression [20]. The assay was designed in 1∶2 serial dilutions, with 10 µM, 5 µM, 2.5 µM, 1.25 µM, 0.625 µM, 0.3125 µM and 0.15625 µM final concentrations, respectively (with the same concentration of the solvent DMSO (1%) in all groups). After 15 days of differentiation, a dose-dependent manner of Troglitazone can be observed through fluorescence microscope, and reflected from the data of real-time PCR and fluorescence spectrophotometer (Fig. 3d–f). From the result of fluorescence spectrophotometer, the EC_50_ of Troglitazone was 0.496 µM, and the maximum effective concentration was 10 µM.

CHIR99021 is a selective inhibitor of glycogen synthase kinase 3 (GSK3), which can mimic stimulation of Wnt signaling pathway in preadipocytes. CHIR99021 inhibits adipogenesis by blocking the induction of CCAAT/enhancer-binding protein α (CEBPα) and PPAR gamma [21]. In this assay, we set up 6 test groups of CHIR99021 in 1∶2 serial dilutions, with 1 µM, 500 nM, 250 nM, 125 nM, 62.5 nM and 31.25 nM final concentrations, respectively (with the same concentration of the solvent DMSO (0.016%) in all groups). In this assay, the data from fluorescence spectrophotometer displayed a dose-dependent manner of CHIR99021, with the EC_50_ of around 206 nM, and the maximum effective concentration was 1 µM (Fig. 3g, 3i). Such dose-dependent manner of CHIR99021 can also be confirmed by real-time PCR analysis of aP2 gene expression (Fig. 3h).

## Discussion

In this study, we introduced a hMSC reporter cell line (aP2-hrGFP hMSCs) as a model applying in small molecules selection in adipogenesis. Firstly, hMSCs were transduced with a lentivector containing aP2-hrGFP. After characterizing the phenotypic and multipotent property of transduced hMSCs, we verified the efficacy of this system through normal adipogenic induction and adipogenic inhibition by Ly294002. Finally, we treated the aP2-hrGFP hMSCs with Troglitazone and CHIR99021 to further prove the feasibility of this system. After serials of tests, we showed that this system is an efficient method for small molecules screening in adipogenesis.

Luciferase reporter assay, antibody label assay and fluorescence protein assay are frequently used by researchers in screening small compounds. Compared with the former two methods, fluorescence protein assay seems to be an appropriate protocol for high-throughput differentiation screening. It circumvents the extra time and money spent during testing, which might be a strength for high content analysis [22]. Furthermore, this approach provides a manner of real-time and noninvasive observation for researchers. Researchers can observe the change of individual cells continuously during the differentiation process and would not need to fix or fractionate cells [12]. Unlike luciferase reporter assay, which measures the total amount of luciferase activity in test wells, fluorescence protein assay reveals data of individual cells in differentiation assay, where the cell population is not homogeneous [23]. Even though the toxic effect on cells of fluorescence protein raises concern about the application of this method, we did not observe obvious cell death during our experiments. To address this issue, we performed cell titer glo assay to test the cell viability of D0 undifferentiated cells and D15 differentiated cells. The result showed no significant difference of cell viability between these two groups. (Fig. S3)

aP2 gene is not only one of the specific markers of mature adipocytes, but also one of the target genes of PPAR gamma [24]. Since the interrelation between PPAR gamma – a central regulator of adipogenesis and aP2 gene expression has been well studied, it is supposed that aP2 promoter-driven reporter gene assay is a more rapid and sensitive procedure than others to identify agents in adipogenesis [25]. From our data, the aP2-driven green fluorescence protein assay is a simple and sensitive protocol for small molecules selection in adipogenesis. After 15 days of adipogenic induction, we detected the fluorescence intensity simply through fluorescence spectrophotometer, with no extra procedures required. More importantly, by comparing with the results of real-time PCR, we proved that this method is as sensitive as real-time PCR that can reflect the exact variation of adipogenic associated genes.

In the future, many improvements can be made in this system to facilitate high-throughput screening. Multi-well plates could improve the efficiency of screening. Also, using the Gateway system, we could change different tissue specific promoters or other types of fluorescence reporter genes, according to the research need. Additionally, this system can be applied in other fields, such as toxicological study. Several research groups have reported that some environmental compounds or contaminants, like TCDD or benzo(a)pyrene, may have toxic effects on adipogenesis in the body through Ah receptor (AhR) [26], [27]. But most of these studies used oil red O staining and real-time PCR as detection tools for analysis, which is either insensitive or time-consuming. By using this fluorescence protein assay, these studies can be carried out very efficiently.

All together, this is a pioneer study of applying fluorescence reporter gene to test small molecules function in adipogenesis, which might become a powerful screening assay facilitating adipocyte biology, drug discovery and toxicological study in the future.

## Materials and Methods

### Cell culture

hMSCs were obtained from Cyagen Biosciences Inc.(Guangzhou, China). The culture medium contained low glucose DMEM (Hyclone, Logan, Utah, USA) supplemented with 10% fetal bovine serum (FBS) (Hyclone), 100 IU/ml penicillin (Hyclone), and 100 µg/ml streptomycin (Hyclone). Medium was changed every 2–3 days. After reaching confluence, cells were detached by 0.25% trypsin for 1–2 min at 37°C and replated for continuous passage.

### Vector construction, lentivirus production and transduction

To generate a pDest_puro_ vector, we used the puromycin resistance encoding sequence to replace both the blasticidin resistance encoding sequence and the bacterial EM7 promoter of lentiviral 2k7_bsd_ vector (kindly provided by Dr. David M. Suter, University of Geneva Medical School, Geneva, Switzerland) [28]. In order to generate entry vectors, we generated rat aP2 promoter (3708 bp) flanked with attB sites from BAC clone CH230-359E6 (provided by Children's Hospital Oakland Research Institute, Oakland, California, USA) using PCR, subsequently cloned the promoter PCR product into pDONR™P4–P1R (Invitrogen, Carlsbad, California, USA) by utilizing the Gateway BP recombination reaction following the product instructions. A hrGFP gene was cloned into pDONR™221 (Invitrogen) by the same method. The resulting vectors, which we named pUp-aP2 and pDown-hrGFP, were then recombined into the pDest_puro_ vector following the protocol for LR recombination reaction using the Gateway LR plus clonase enzyme mix (Invitrogen) to construct the expression lentiviral vector, designated as pLV/Final-puro-aP2-hrGFP.

The lentiviral particles were prepared by transient cotransfection of 293FT cells with the lentiviral vector pLV/Final-puro-aP2-hrGFP and ViraPower™ Lentiviral packaging mix (Invitrogen) using Lipofectamine 2000 (Invitrogen). Three days after transfection, viral particles were harvested from the medium, filtered through 0.45-µm pore-sized polyethersulfone membrane, and concentrated by ultracentrifugation (50,000 g, for 120 minutes at 4°C).

For lentiviral transduction, hMSCs were washed with PBS and dissociated to single cells by 0.25% trypsin and were replated with lentiviral particles and 5 µg/ml polybrene (Sigma, St Louis, Missouri, USA). The medium was changed to fresh culture medium after infection for 12 hours. After twice transduction, puromycin (Sigma) was added to the culture medium at a concentration of 1–5 µg/ml and maintained for 5 days.

### Fluorescence-activated cell sorting analysis

Membrane antigen expression of untransduced and transduced hMSCs was determined by flow cytometry. Cells were harvested and incubated with monoclonal antibodies against human antigens including CD29, CD34, CD44, CD45, CD73, and CD166 (all from BD Biosciences, Palo Alto, CA, USA). Irrelevant isotype-identical antibody (BD Biosciences) served as negative control. Samples were analyzed by collecting 10,000 events using Cell-Quest® software (BD Biosciences).

### Osteogenic and chondrogenic differentiation of transduced hMSCs

For osteogenic differentiation, transduced hMSCs were seeded in six-well plate at the density of 5×10^3^/cm^2^. When the cells reached 80% conflucence, culture medium was replaced with the osteogenic medium containing 10^−7^ M dexamethasone (Sigma), 10 mM β-glycerophosphate (Sigma), and 50 µg/ml vitamin C (Sigma). After 14 days, osteogenic differentiation was evaluated by alizarin red S staining.

Chondrogenic differentiation was induced on cell pellet culture system as previously described [29]. In brief, 500,000 transduced hMSCs were suspended in one 15 ml conical tube with 2 ml induction medium including DMEM containing 3% FBS, 10 ng/ml TGF-β3 (PeproTech, NJ, USA), 6.25 µg/mL insulin (Sigma), 6.25 µg/mL transferrin (Gibco), 1.25 µg/mL BSA (Sigma), and 1 mM pyruvate (Sigma), The medium was replaced every 3 days for 21 days.

### Adipogenic differentiation of transduced hMSCs

To induce adipogenic differentiation, one-day post-confluent transduced hMSCs were cultured in DMEM containing 10% FBS, 10 µg/ml insulin, 1 µM dexamethasone, 200 nM indomethacin (Sigma) and 0.5 mM 3-isobutyl-1-methylxanthine (Sigma). The medium was replaced every 3 days for 15 days. For verification assays, transduced hMSCs were seeded in 96-well plates. On differentiation, LY294002 (Cayman Chemical, Ann. Arbor, MI, USA) (dissolved in DMSO, storage concentration is 50 mM) or CHIR99021 (Stemgent, Cambridge, MA, USA) (dissolved in DMSO, storage concentration is 6 mM) was added in the induction medium, while Troglitazone (Cayman Chemical, Ann Arbor, MI, USA) (dissolved in DMSO, storage concentration is 1 mM) was added in the induction medium without indomethacin (DMSO as solvent control). During differentiation, we observed the hrGFP expression through fluorescence microscope every three days. hrGFP intensity was detected by Fluorescence Spectrophotometer (Tecan Trading AG, Switzerland) at the excitation wavelength of 485 nm and emission wavelength of 535 nm. For each molecule, three independent experiments were performed and there were six replicate wells for each concentration. Each well was detected for three times and data were collected symmetrical from four different points in a well.

### Immunochemistry and histochemistry

For immunofluorescence studies, differentiated transduced cells were incubated with primary antibody against aP2 (Abcam, Cambridge, UK) over night at 4°C. The secondary antibody, goat Cy3-conjugated anti-mouse (Jackson, West Grove, Pennsylvania, USA), was added at room temperature for 1 h in the dark. Nucleus was counterstained with Hoechst33342 (Sigma). Undifferentiated cells were used as negative controls.

For oil red O staining, adipogenic differentiated cells were fixed and incubated with oil red O (Sigma) for 30 min. For alizarin red S staining of calcium, osteogenic differentiated cells were fixed and incubated with alizarin red S (Sigma) for 20 min. For assessment of chondrogenic differentiation, tissue sections of formalin-fixed and paraffin-embedded samples were incubated with anti-collagen II antibody (1∶200, Chemicon), overnight at 4°C. After rinsing with PBS, slides were incubated for 10 min at room temperature with biotin-conjugated secondary antibodies, followed by incubation with streptavidin-conjugated peroxidase working solution for 10 min. Subsequently, sections were stained for 15–30 min with 3-amino-9-ethylcarbazole (AEC), counterstained with Mayer's haematoxylin. Negative controls were prepared by substituting PBS for primary antibody.

### Real-time PCR analysis

Total RNA was extracted from undifferentiated and differentiated transduced hMSCs with Trizol reagent (Invitrogen). For eliminating any contaminating genomic DNA, the total RNA was subjected to DNase I (Fermentas, Maryland, USA) according to the directions of the manufacturer. Reverse transcription was carried out using Murine Leukemia Virus reverse transcriptase (Fermentas) and oligo-dT primers (Fermentas) according to the manufacturer's instruction.

Real-time PCR was performed with the Bio-Rad CFX96 Detection System (Bio-Rad, Hercules, CA, USA) and QuantiTect SYBR Green PCR Master Mix (Qiagen, Valencia, CA, USA). All reactions were run in triplicate. The primers used in this study were as follows: GAPDH (forward), 5′-GAAGGTGAAGGTCGGAGTC-3′; GAPDH (reverse), 5′-GAAG ATGGTGATGGGATTTC-3′; PPAR gamma (forward), 5′-CGAGAAGGAGAAGCTGT TGG-3′; PPAR gamma (reverse), 5′-TCAGCGGGAAGGACTTTATGTATG-3′; aP2 (forward), 5′-AGCACCATAACCTTAGATGGGG-3′; aP2 (reverse), 5′-CGTGGAAGTGACGCCTTTCA-3′. GPDH (forward), 5′-AGGAAGACATTGGAGGCAAAAA-3′; GPDH (reverse), 5′-GCAGCCTGGACCACATCTG-3′;

### Cell Titer Glo Assay

Undifferentiated and differentiated cells were plated in 96-well opaque plate (10^5^cells/well). Cell Titer Glo assay was performed according to the manufacturer's instruction (Promega, San Luis Obispo, CA, USA), and measured by Spectrophotometer (Tecan Trading AG, Switzerland). Data were collected from three independent experiments.

### Statistical analysis

Data are presented as means ± SDs, and the one-way ANOVA test was used for testing statistical significance.

## Supporting Information

Figure S1 a, Vector construction of pLV/Final-bsd-2.3Col-RFP by the multisite gateway technology. This 2.3Col promoter was kindly provided by Professor Peng Liu [Yin D, et al. 2009]. We used PCR to generate attB-flanked 2.3Col promoter, and subsequently cloned the promoter PCR product into pDONRTMP4-P1R (Invitrogen) by Gateway BP recombination reaction. The RFP gene was cloned into pDONRTM221 (Invitrogen) by the same method. The resulting vectors, named pUp-2.3Col and pDown-RFP respectively, were then recombined into the pDestbsd vector by LR recombination reaction to construct the expression lentiviral vector, designated as pLV/Final-bsd-2.3Col-RFP; b, Fluorescence protein expression of aP2-hrGFP/2.3Col-RFP hMSCs in different days of differentiation. These cells were induced to differentiate with dual lineage promoting medium consisting of a mixture of adipogenic and osteogenic medium (V/V; 1/1). Along the 15 days of induction, the reporter gene expression was observed every three days through the fluorescence microscope. Bar = 50 µm.
(2.74 MB TIF)

Figure S2 a, Normalized mRNA expression of aP2 gene determined by real-time PCR during adipogenesis; b, Fluorescence intensity detection of aP2-hrGFP hMSCs by fluorescence spectrophotometer during normal adipogenic induction; c, Cell Titer Glo assay of D0, D15 and D18 adipogenic differentiated cells; d and e, Oil red O staining of D15 and D18 differentiated cells. Data are presented as mean ± SD of three independent experiments, and the p-values in the graph show the statistical significance of the difference between each test day and the D0 control group. (★p<0.05; ★★p<0.01); Bar = 50 µm.
(2.35 MB TIF)

Figure S3 Cell Titer Glo assay of D0 and D15 adipogenic differentiated cells. Data are presented as mean ± SD of three independent experiments, and the p-values in the graph show the statistical significance of the difference between D0 and D15 differentiated cells. (★p<0.05; ★★p<0.01).
(4.10 MB TIF)
