# Supplementary figures and images for: A Stem Cell-Based Tool for Small Molecule Screening in Adipogenesis
Source: PLoS One. 2010 Sep 27;5(9):e13014. doi: 10.1371/journal.pone.0013014 (PMC2946407; doi:10.1371/journal.pone.0013014)

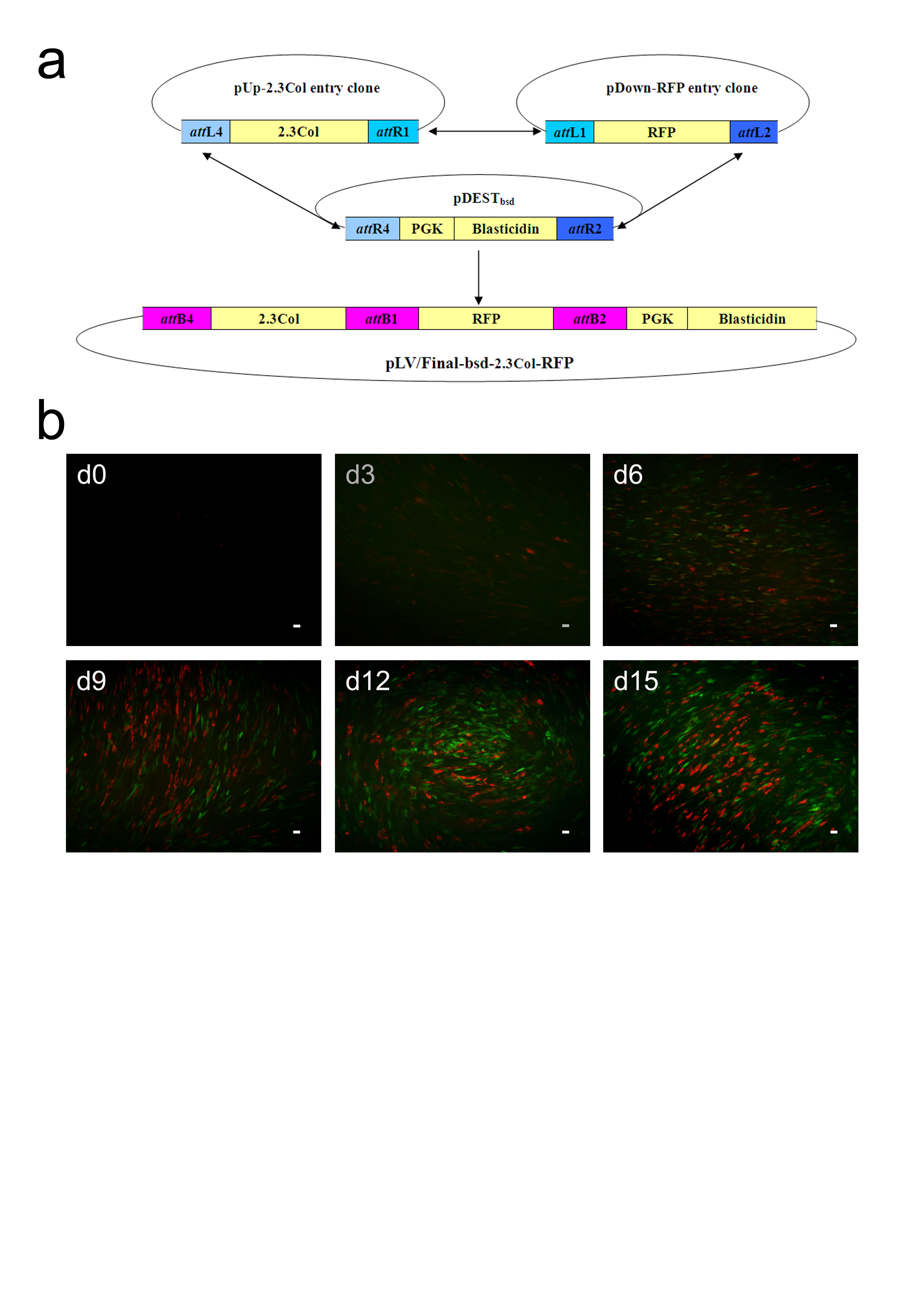

Supplement: Figure S1 — a, Vector construction of pLV/Final-bsd-2.3Col-RFP by the multisite gateway technology. This 2.3Col promoter was kindly provided by Professor Peng Liu [Yin D, et al. 2009]. We used PCR to generate attB-flanked 2.3Col promoter, and subsequently cloned the promoter PCR product into pDONRTMP4-P1R (Invitrogen) by Gateway BP recombination reaction. The RFP gene was cloned into pDONRTM221 (Invitrogen) by the same method. The resulting vectors, named pUp-2.3Col and pDown-RFP respectively, were then recombined into the pDestbsd vector by LR recombination reaction to construct the expression lentiviral vector, designated as pLV/Final-bsd-2.3Col-RFP; b, Fluorescence protein expression of aP2-hrGFP/2.3Col-RFP hMSCs in different days of differentiation. These cells were induced to differentiate with dual lineage promoting medium consisting of a mixture of adipogenic and osteogenic medium (V/V; 1/1). Along the 15 days of induction, the reporter gene expression was observed every three days through the fluorescence microscope. Bar = 50 µm. (2.74 MB TIF) [file pone.0013014.s001.tif]

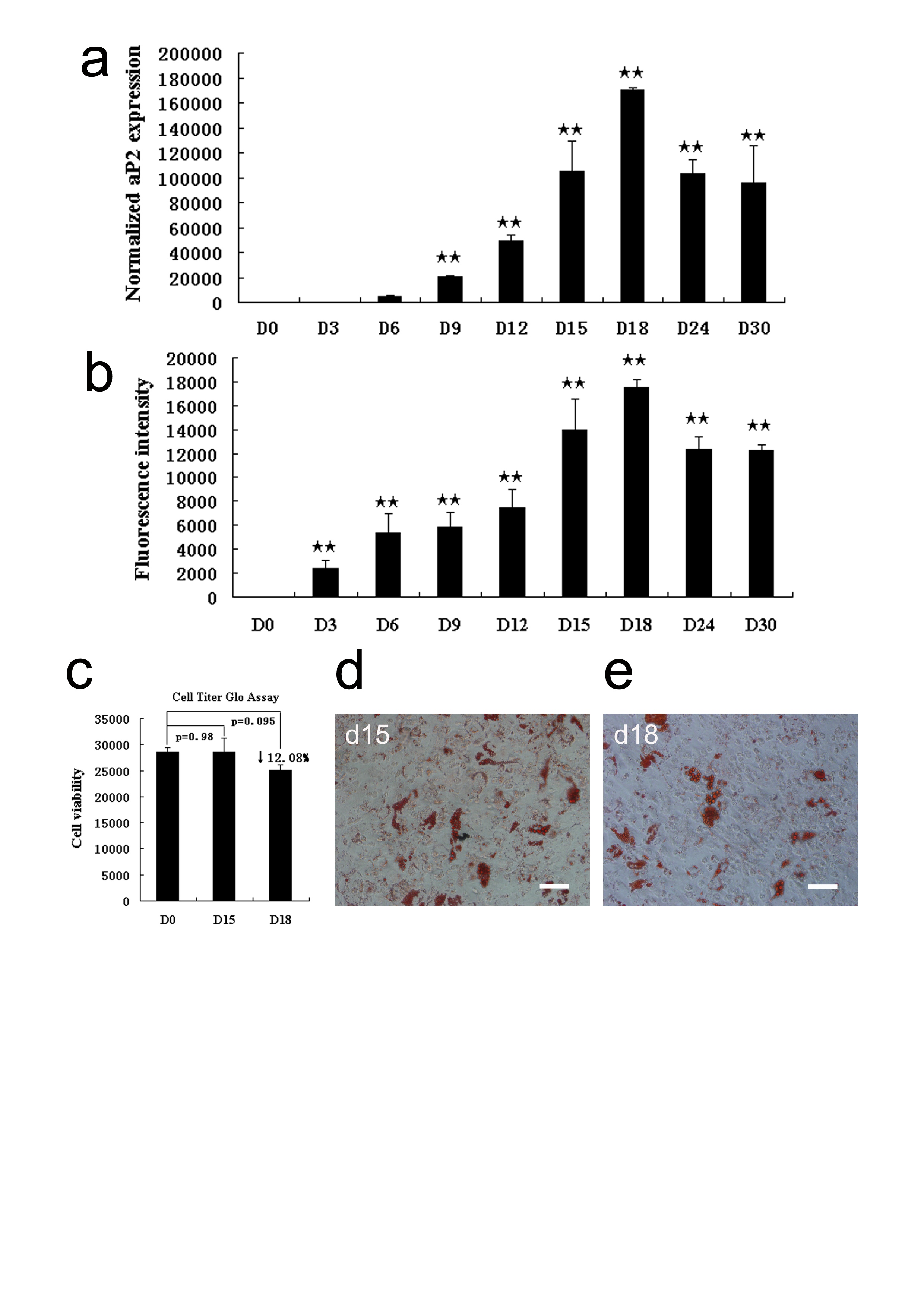

Supplement: Figure S2 — a, Normalized mRNA expression of aP2 gene determined by real-time PCR during adipogenesis; b, Fluorescence intensity detection of aP2-hrGFP hMSCs by fluorescence spectrophotometer during normal adipogenic induction; c, Cell Titer Glo assay of D0, D15 and D18 adipogenic differentiated cells; d and e, Oil red O staining of D15 and D18 differentiated cells. Data are presented as mean ± SD of three independent experiments, and the p-values in the graph show the statistical significance of the difference between each test day and the D0 control group. (★p<0.05; ★★p<0.01); Bar = 50 µm. (2.35 MB TIF) [file pone.0013014.s002.tif]

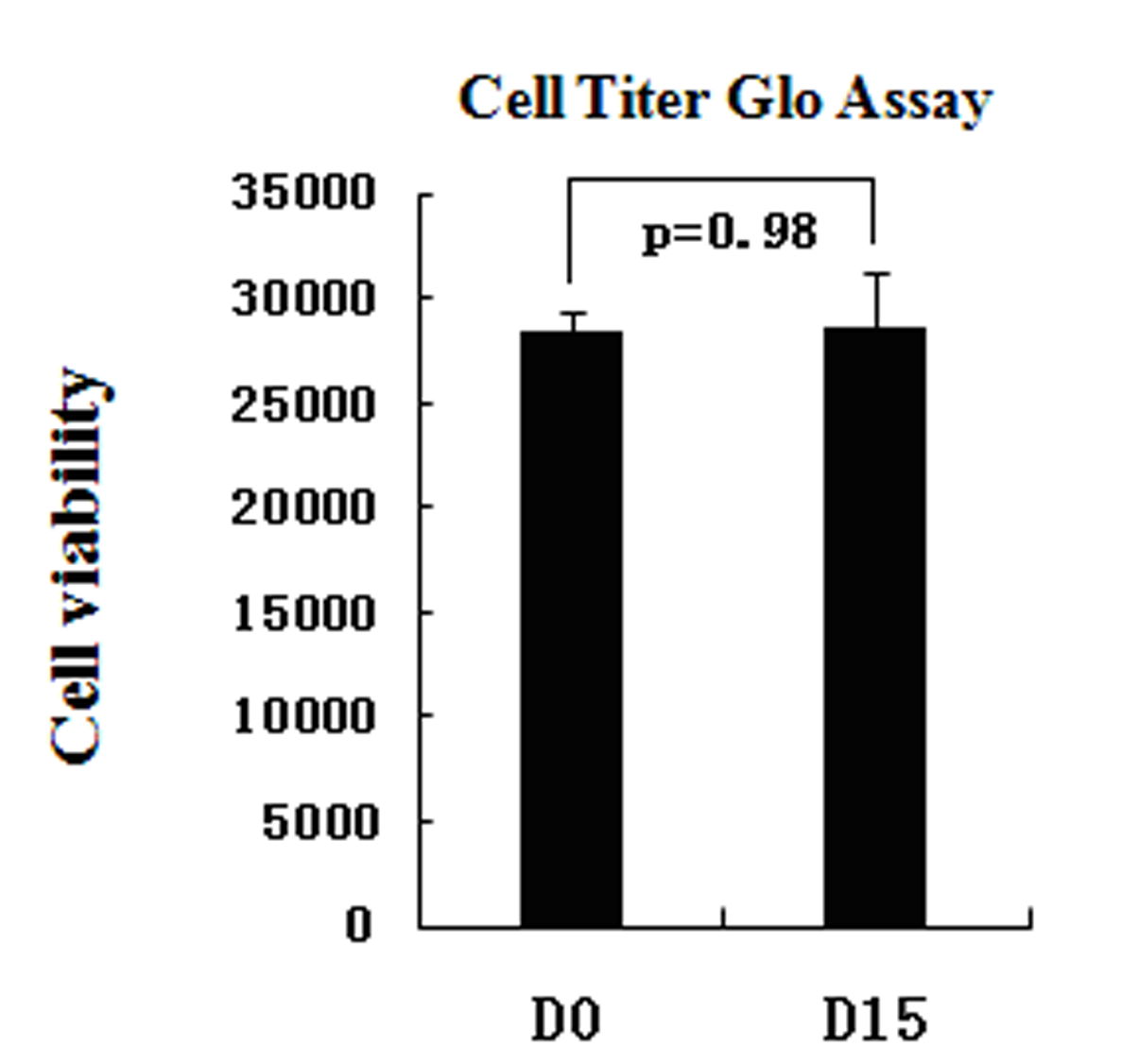

Supplement: Figure S3 — Cell Titer Glo assay of D0 and D15 adipogenic differentiated cells. Data are presented as mean ± SD of three independent experiments, and the p-values in the graph show the statistical significance of the difference between D0 and D15 differentiated cells. (★p<0.05; ★★p<0.01). (4.10 MB TIF) [file pone.0013014.s003.tif]
